# Supplementary material for: BR-bodies link RNA homeostasis to stress tolerance and intracellular fitness in Brucella
Source: bioRxiv. 2026 Jul 13:2026.07.11.737834. Preprint. [Version 1] doi: 10.64898/2026.07.11.737834 (PMC13405055; doi:10.64898/2026.07.11.737834)
Supplement: Supplementary file 1 [file NIHPP2026.07.11.737834v1-supplement-2.pdf]

bioRxiv preprint doi: https://doi.org/10.64898/2026.07.11.737834; this version posted July 13, 2026. The copyright holder for this preprint (which was not certified by peer review) is the author/funder, who has granted bioRxiv a license to display the preprint in perpetuity. It is made available under a CC-BY 4.0 International license.

Supplementary Figures

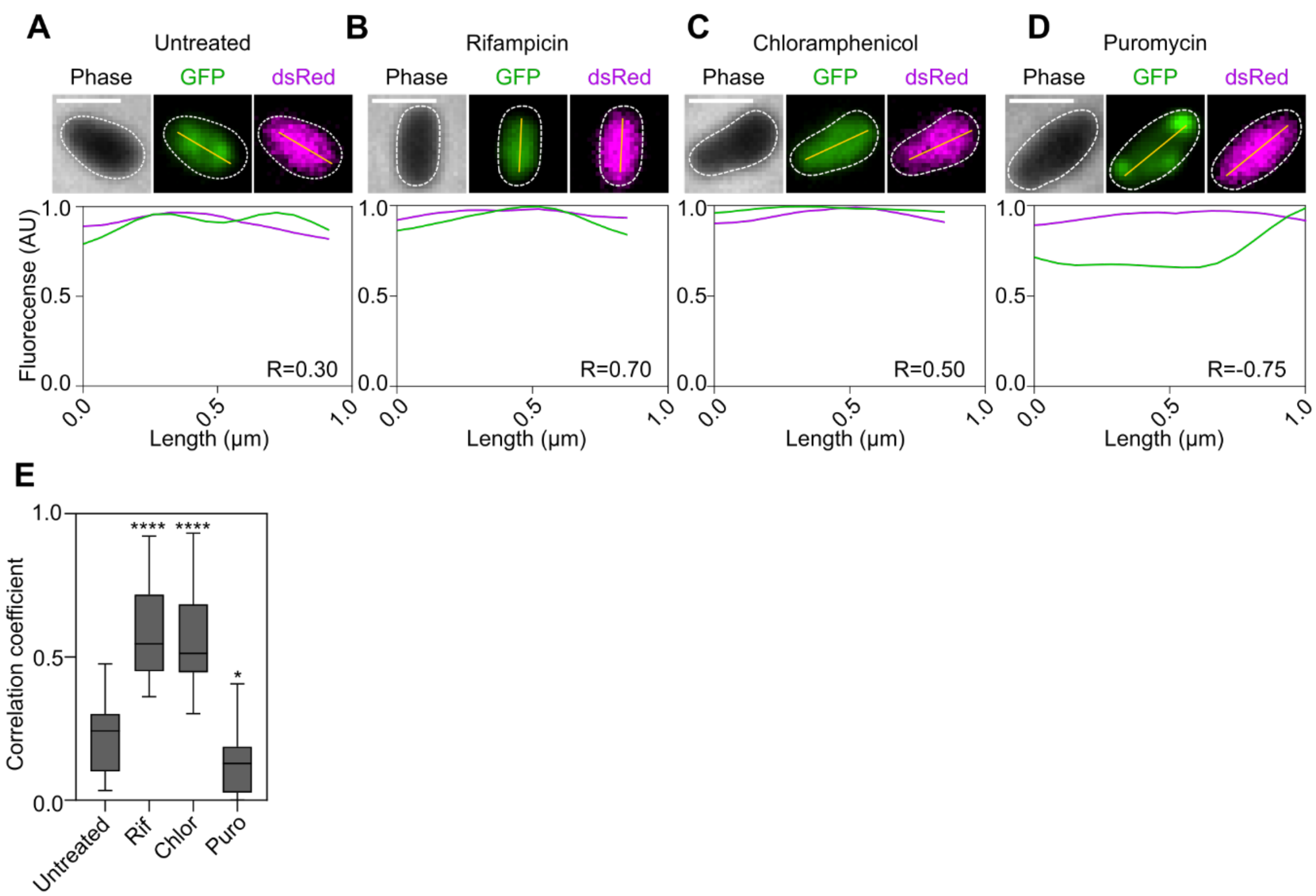

Figure S1: Line trace analysis of *rne*-GFP relative to cytoplasmic dsRed control. A-D) Phase-contrast and fluorescence microscopy images of dual-labeled *B. ovis rne*-GFP cells expressing full-length RNase E-GFP (green) together with a constitutive *glmS*::dsRed marker (magenta) in untreated **(A)**, rifampicin (100 µg/mL, 30 min) treated **(B)**, chloramphenicol (100 µg/mL, 15 min) treated **(C)**, or puromycin (150 µg/mL, 30 min) treated **(D)** cells. The yellow line indicates the transect used to generate the fluorescence intensity profiles shown below each image; line profiles were quantified in Fiji (ImageJ). Scale bars, 1 µm. (**E)** Colocalization between GFP and dsRed signals, expressed as Pearson correlation coefficients calculated from 60 cells across three biological replicates for untreated and drug treated conditions. Bars show mean values and error bars indicate standard deviation. (*, p< 0.05; ****, p < 0.0001, two-tailed *t* test with unequal variance).

bioRxiv preprint doi: https://doi.org/10.64898/2026.07.11.737834; this version posted July 13, 2026. The copyright holder for this preprint (which was not certified by peer review) is the author/funder, who has granted bioRxiv a license to display the preprint in perpetuity. It is made available under a CC-BY 4.0 International license.

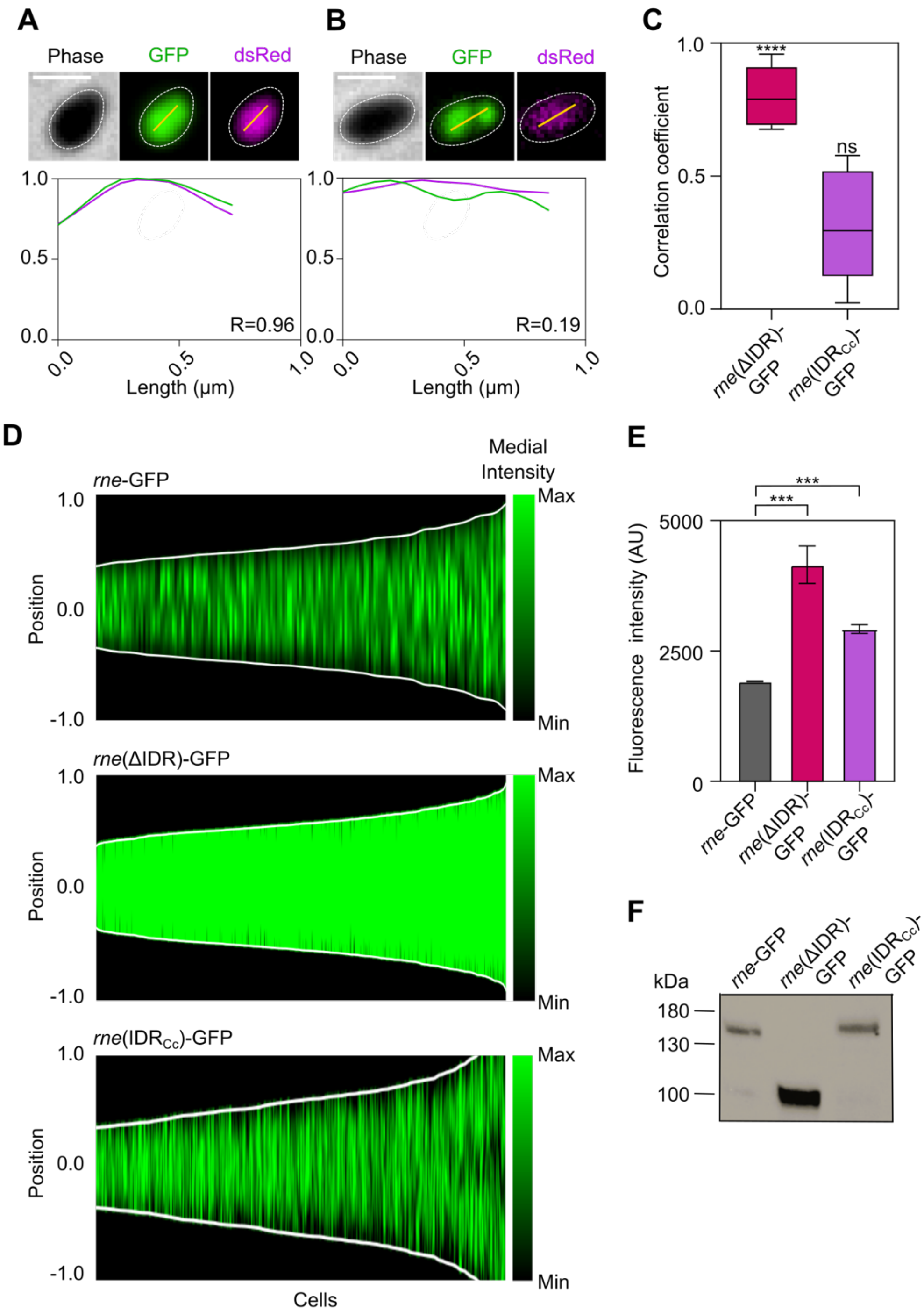

Figure S2: *rne*(ΔIDR)-GFP and *rne*(IDR_{Cc})-GFP expression and localization. A) Phase-contrast and fluorescence images of dual-labeled *rne*(ΔIDR)-GFP cells expressing RNase EΔIDR-GFP (green) together with a constitutive *glmS*::dsRed marker (magenta). The yellow line indicates the transect used to generate the fluorescence intensity profiles shown below the image; line profiles were quantified in Fiji (ImageJ). **B)** Phase-contrast and fluorescence images of *rne*(IDR_{Cc})-GFP cells expressing the chimeric *rne* (the *C. crescentus* IDR replacement, *rne*(IDR_{Cc})-GFP), as in (A). **C)** Colocalization between GFP and dsRed signals,

bioRxiv preprint doi: https://doi.org/10.64898/2026.07.11.737834; this version posted July 13, 2026. The copyright holder for this preprint (which was not certified by peer review) is the author/funder, who has granted bioRxiv a license to display the preprint in perpetuity. It is made available under a CC-BY 4.0 International license.

expressed as Pearson correlation coefficients calculated from 30 cells from three biological replicates for *rne*(ΔIDR)-GFP and *rne*(IDR_{Cc})-GFP. Bars show mean values and error bars indicate standard deviation. (****, $p < 0.0001$; two-tailed t test with unequal variance). **D**) Demographs of GFP fluorescence intensity along the medial axis of individual *rne*-GFP cells (n = 171 cells), *rne*(ΔIDR)-GFP cells (n = 715 cells), and *rne*(IDR_{Cc})-GFP cells. Cells are ordered by increasing cell length; intensity is plotted along a normalized cell axis. **E)** Total GFP fluorescence intensity per cell (arbitrary units), reporting relative RNase E protein abundance, in *rne*-GFP cells, *rne*(ΔIDR)-GFP cells, and *rne*(IDR_{Cc})-GFP cells; bars show mean ± standard deviation (***, $p < 0.001$; two-tailed t test with unequal variance). **F)** Western blot of *rne*-GFP, *rne*(ΔIDR), and *rne*(IDR_{Cc}) strains; molecular-weight markers (kDa) are indicated at left.

bioRxiv preprint doi: https://doi.org/10.64898/2026.07.11.737834; this version posted July 13, 2026. The copyright holder for this preprint (which was not certified by peer review) is the author/funder, who has granted bioRxiv a license to display the preprint in perpetuity. It is made available under a CC-BY 4.0 International license.

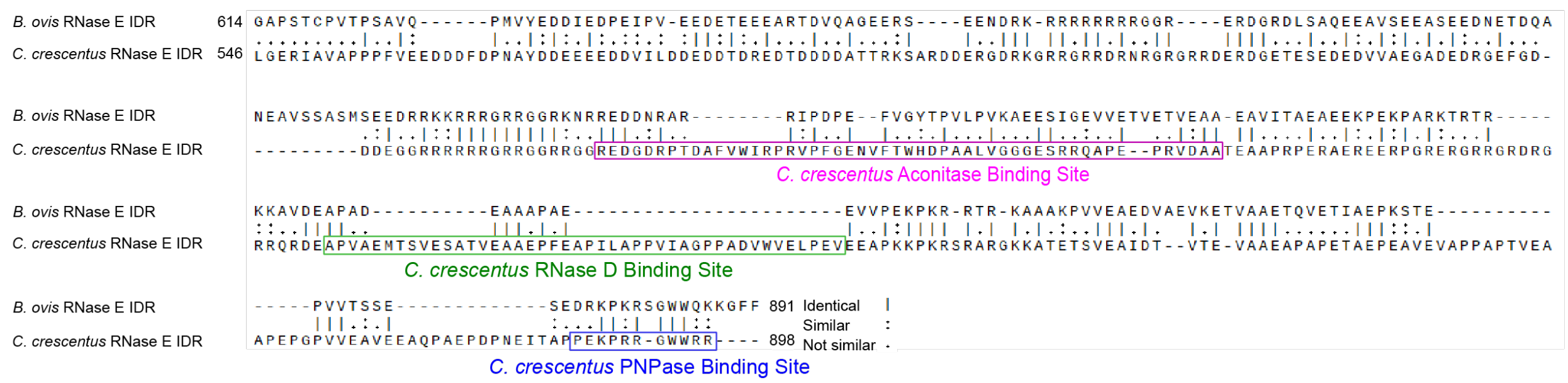

Figure S3: *B. ovis* RNase E IDR is highly divergent from the *C. crescentus* RNase E IDR. The *B. ovis* RNase E IDR shares only 30.29% overall sequence identity with the *C. crescentus* RNase E IDR (Needleman-Wunsch global alignment, alignment matrix: BLOSUM62, gap open penalty:10.0, gap extend penalty: 1.0). Known *C. crescentus* degradosome binding sites within the IDR also show low sequence identity when aligned to the *B. ovis* IDR: RNase D binding site (40%), aconitase binding site (39%), and PNPase binding site (60%) (Smith-Waterman local alignment, alignment matrix: BLOSUM62, gap open penalty:10.0, gap extend penalty: 1.0).

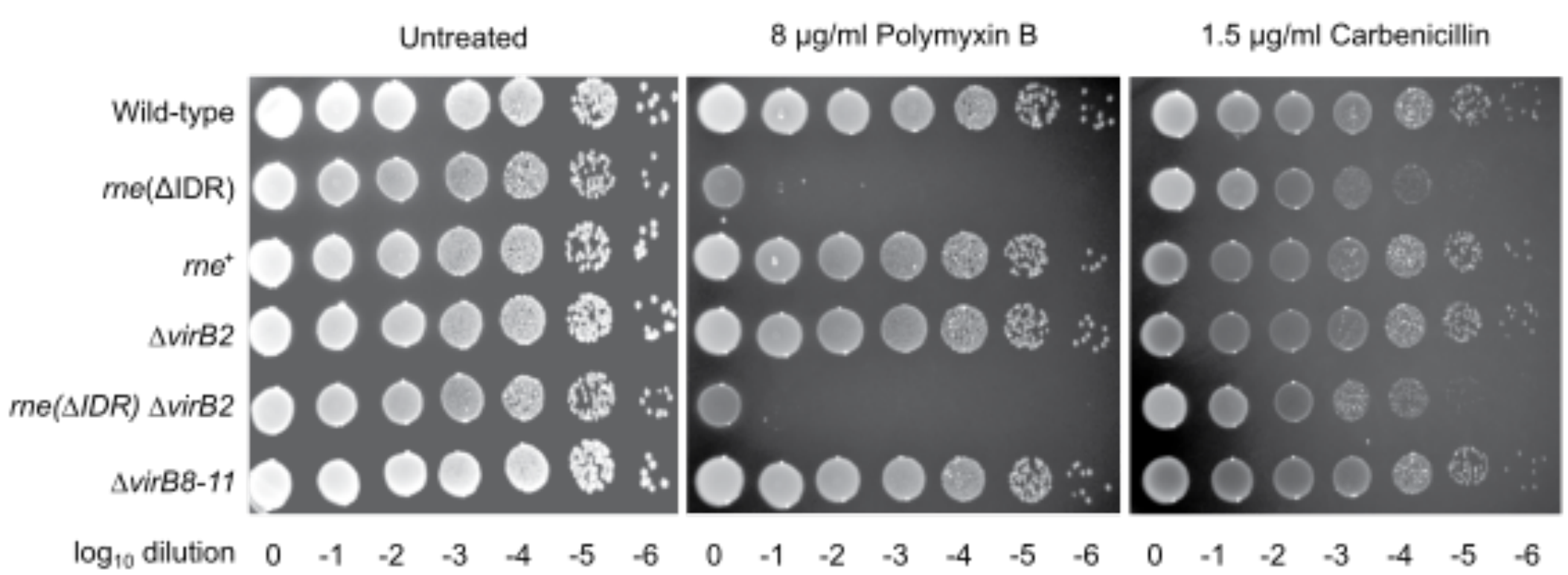

Figure S4: *virB* mutants do not impact stress sensitivity. Spot dilution assays of wild type, *rne*(ΔIDR), *rne*+, Δ*virB2*, *rne*(ΔIDR) Δ*virB2,* and *ΔvirB8-11* for stress sensitivity. Strains on TSA blood agar were resuspended, normalized to OD_{600} = 0.3, serially diluted 10-fold, and spotted (5 µL) onto TSA blood agar plates lacking stressor or supplemented with polymyxin B (8 µg/mL), carbenicillin (1.5 µg/mL). Plates were incubated at 37°C and imaged after 72-96 hours. Dilution plating experiments were repeated at least three times (n=3) in technical triplicate, and one representative experiment is shown.

bioRxiv preprint doi: https://doi.org/10.64898/2026.07.11.737834; this version posted July 13, 2026. The copyright holder for this preprint (which was not certified by peer review) is the author/funder, who has granted bioRxiv a license to display the preprint in perpetuity. It is made available under a CC-BY 4.0 International license.

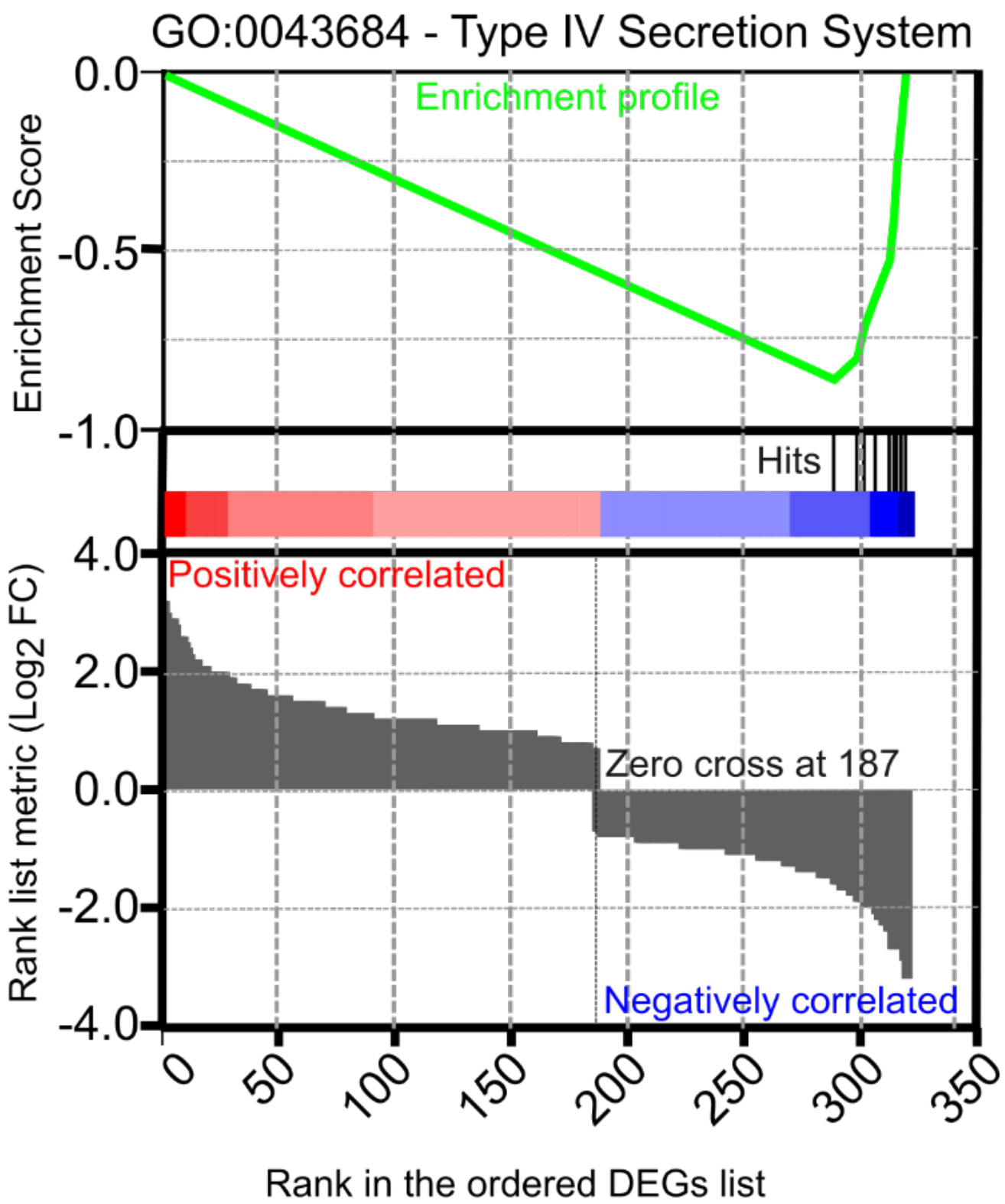

Figure S5: Annotated type IV secretion system (T4SS) components are enriched at the bottom of the ranked gene list and are collectively downregulated in the *rne*(ΔIDR) mutant. Gene Set Enrichment Analysis (GSEA) enrichment plot for GO:0043684 (type IV secretion system) across the ranked gene list. The green curve represents the running enrichment score, and vertical tick marks indicate the positions of genes annotated with the type IV secretion system GO term within the ranked list. The color bar and ranked-metric panel show the distribution of $\log_2$ fold changes used to rank the genes.
